# Multi-criteria inventory classification considering demand stability

**DOI:** 10.1038/s41598-026-42590-0

**Published:** 2026-03-30

**Authors:** Changwen Wang, Guolong Ning

**Affiliations:** State Key Laboratory of Advanced Design and Manufacturing for Vehicle, Hunan University, No. 2 South Lushan Road, Yuelu District, Changsha, 410082 Hunan China

**Keywords:** Inventory classification, Multi-criteria, Inventory criticality, Demand stability, ABC, Mechanical engineering, Computational science, Applied mathematics

## Abstract

This paper focuses on the demand stability criterion for inventory classification. Unstable demand may lead to inventory obsolescence or emergency purchasing. A case study of multi-criteria inventory classification (MCIC) for a tunnel boring machine manufacturer is conducted. An MCIC method based on a classical MCIC model is proposed. The proposed method first evaluates demand criticality using the mean, standard deviation and range of demand. And then, the proposed method calculates the final criticality scores for Stock Keeping Units (SKUs) based on demand criticality and other criteria, e.g., unit price or lead-time. Finally, SKUs are ranked in descending order and classified by the ABC principle. The classification results can draw the attention of inventory managers to the unstable SKUs. The inventory performance analysis shows that inventory classification considering demand stability can achieve higher fill rates at lower cost.

## Introduction

Inventory management plays an important role in Industry 4.0 manufacturing^1^. An appropriate inventory control strategy can provide parts on time while reducing the inventory cost. In the after-service market, effective inventory control of spare parts can ensure operational efficiency and timely maintenance of equipment.

For many industries, such as vehicle manufacturing, household appliance production, and aircraft assembly, unstable demand and managing a large number of SKUs are two key challenges for inventory managers. Most complex products are assembled from thousands of materials or parts. Manufacturers often manage massive SKUs to guarantee the production or stock enough spare parts for after-sales services. It is a great challenge for inventory managers to specify an inventory policy for each SKU. Moreover, demand for SKUs is often unstable. Inventory managers often encounter situations in which unexpected, enormous demand arises, or inventory suddenly becomes obsolete. They have to maintain high inventory levels or high purchasing frequencies to cope with the impact of unstable demand.

To cope with the first challenge, a common approach is to classify items according to given criteria. Then inventory managers could apply the same policy to items in a specific class. It can reduce inventory cost if inventory managers adopt appropriate inventory strategies for each class^2–5^. Relevant and effective criteria are indispensable for inventory classification. Inadequate selection of criteria may lead to suboptimal classification results^6^. A single criterion cannot conduct a comprehensive evaluation of SKUs. Thus, the MCIC is an effective method for inventory managers. To address the unstable demand challenge, improving forecast accuracy is paramount. However, the benefits of improving demand forecast accuracy are limited due to the volatility of future demand. In such a scenario, incorporating demand stability as a criterion in MCIC is an effective approach.

The existing research on MCIC mainly focuses on method innovation. The demand stability criterion is almost irrespective when scholars select criteria. The inventory measurements under the demand criterion are usually calculated using mean demand or demand volume, which cannot reflect the stability of demand. In some papers, measurements are calculated based on annual dollar usage. But the demand volume can be calculated from annual dollar usage and unit price, which are usually used concurrently^7,8^.

This paper aims to propose an efficient method that inventory managers can conveniently use in a spreadsheet. The proposed method first evaluates demand criticality based on statistical measures of historical demand that reflect demand size and stability. And then, the final inventory criticality scores are evaluated using the proposed method. Both phases are by the Ng-model^8^. Thus, the proposed method is called the Double Ng-model (D-Ng-model). In the process of classifying SKUs, the demand stability is considered, and the weights are endogenous. The mean, standard deviation, and range of demand are used to calculate the scores for the demand stability criterion. To evaluate the performance of the proposed MCIC method, an inventory classification study conducted by a manufacturer in China is presented. The results indicate that the proposed method is effective. The empirical investigation results demonstrate that inventory managers can achieve higher fill rates at a lower cost by using the proposed method.

The contributions of the proposed method are as follows:

1. An efficient MCIC method is proposed;
2. The proposed method can be utilized just on a spreadsheet;
3. The criterion of demand stability is considered for classifying SKUs;
4. The demand range criterion is introduced to evaluate demand stability.

## Literature review

In the literature, the ABC method is widely used in practice and academia. By the Pareto principle, the top 20% of items in class A are the most important, the bottom 50% of items in class C are less important, and the rest in class B are moderately important^9^. Inventory systems can achieve better performance through the ABC classification method.

Because the dimensions and weights of the criteria differ, inventory managers should aggregate the scores into a single final score for classifying items. Many methods have been developed to solve this problem, such as Analytic Hierarchy Process (AHP)^10^, Data Envelopment Analysis (DEA)^11^, Artificial Intelligence (AI)^12,13^, and DEA-like models^7,8,14,15^.

The AHP method is helpful for MCIC. Most researchers combine AHP with other methods to make the MCIC more effective. Cakir and Canbolat^10^ first adopted the fuzzy concept in the AHP by using fuzzy linguistic comparisons to obtain uncertain judgments from inventory managers. The authors also develop a web-based system for implementing the fuzzy AHP model. Chu et al.^16^ combined the fuzzy method with the ABC method to easily incorporate managers’ experience, knowledge, and judgment for classifying inventory items. Hadi-Vencheh and Mohamadghasemi^17^ integrated the fuzzy theory, AHP, and DEA methods for MCIC problems. Lolli et al.^18^ introduced a hybrid method that combines the AHP and K-means methods, and a variant called AHP-K-Veto. Ishizaka et al.^19^ presented a DEA Sort approach for sorting problems in inventory classification. The proposed method applies AHP to determine the criterion weights and then uses a variant of DEA model to calculate the priorities of inventory items. Nariswari et al.^20^ applied the AHP method to improve the management efficiency of a large-scale aircraft maintenance and repair firm. Yigit and Esnaf^21^ proposed a three-phase MCIC method. The proposed method first calculates the criteria weights using AHP. Rodríguez et al.^22^ used the AHP fuzzy TOPSIS tool for multi-criteria ABC inventory classification in a medium-sized company. Torre et al.^23^ developed a decision support system using the AHP method in the steel industry. Note that, all AHP-based methods require subjective judgments from inventory managers.

Unlike AHP, the criteria weights are endogenous in the DEA-like models. Ramanathan^7^ first proposed a simple linear programming model (R-model) similar to the DEA model. The maximization objective function is the optimal inventory score for each item, and the model should be solved repeatedly for each item. Ng^8^ argued that the processing time could be very long by the R-model when classifying thousands of items. The paper then proposed another linear optimization model (Ng-model). By a transformation, the Ng-model can be solved without a linear optimizer. Zhou and Fan^14^ discovered that an item with a high measurement under an unimportant criterion may be classified into class A by the R-model and proposed a model (ZF-model). Hadi-Vencheh^15^ thought that the score of one item is independent of the Ng-model and then presented a nonlinear programming model (H-model) to calculate the total score of each item. Babai et al.^24^ conducted an empirical investigation of four DEA-like models, the Ng-model^8^, R-model^7^, ZF-model^14^ and H-model^15^. The results show that the Ng-model presents the lowest inventory cost. Considering the combined service-cost performance, although the Ng-model is not the best, the scores of the four DEA-like models are all over 97% and are approximate. Torabi et al.^25^ proposed a linear programming model to solve MCIC problems under both quantitative and qualitative criteria. As the model is a mixed objective-subjective approach, Hatefi et al.^26^ introduced an improvement model that is a completely objective approach. Park et al.^27^ introduced a cross-evaluation-based weighted linear optimization model that combines a cross-efficiency evaluation method with the R-model. Tavassoli and Saen^28^ Tavassoli and Saen presented a new approach to categorize inventory items based on stochastic data and the nature of the criteria, and proposed a new stochastic mixed integer programming model.

The AI methods can also solve MCIC problems. Partovi and Anandarajan^29^ adopt Artificial Neural Networks (ANNs) for MCIC problems and apply the method to a pharmaceutical company. The proposed method uses backpropagation and Genetic Algorithms (GAs) as learning methods. Kartal et al.^30^ adopt simple-additive weighting, AHP, and VIKOR methods for ABC analyses. And then the authors apply ANN and support vector machine (SVM) algorithms to classify items. Lopez-Soto et al.^31^ also use the ANN method for the MCIC ABC classification. The proposed ANN method adds neurons to the network hidden layer by a randomized greedy strategy. Cui et al.^32^ introduced the back-propagation neural network for MCIC. Yang et al.^13^ presented a three-phase method for classifying spare parts. The method applies the transfer learning theory using deep convolutional neural networks. However, the AI methods are not easily understood by inventory managers and require substantial training data. Qaffas et al.^33^ proposed a semi-supervised explainable approach based on both semi-supervised clustering and explainable artificial intelligence for MCIC. Keskin and Taskin^34^ proposed a new hybrid method combining 9 AI methodologies for MCIC. The AI methods are not easily understood by inventory managers and are not conveniently applied in the manufacturing or after-sales markets.

There is another direction for MCIC research based on inventory performance. Mohammaditabar et al.^35^ build a multi-objective optimization model that simultaneously classifies inventories and determines the optimal policy. One objective of the proposed method is to minimize the total cost of the inventory system. Millstein et al.^36^ introduced a mixed-integer linear program with the objective of maximizing total net profit. The optimization model simultaneously optimizes the number of inventory classes, service levels, and the assignment of items to classes. Sheikh-Zadeh and Rossetti^37^ present a classification optimization method for a two-echelon inventory system based on inventory performance. Sheikh-Zadeh et al.^38^ introduce a mixed-integer and nonlinear optimization model for multi-echelon repairable parts. Sheikh-Zadeh et al.^39^ develop the performance-based inventory classification method for a multi-item, multi-echelon inventory system. Kuo and Jiang^40^ proposed a mathematical model to maximize fill rate by classifying all product items into four groups. Note that, these performance-based optimization models must be solved by an optimizer.

As to other methods, Keshavarz Ghorabaee et al.^41^ proposed a new evaluation method based on the Distance from Average Solution (EDAS) for MCIC problems. Simsek et al.^42^ employed the EDAS method to undertake a multi-criteria inventory classification process for a community pharmacy. Wu^43^ proposed a Parsimoniously Interactive Multi-attribute Rating (PIMAR) method for large-scale inventory classification. Xu et al.^44^ proposed a group decision making model with individual preferences to investigate the multicriteria ABC inventory classification problem. Hadi-Vencheh et al.^45^ proposed a Hybrid TOPSIS-Alternative Factor Extraction Approaches (TOPSIS-AFEA) for MCIC. Cakmak and Guney^46^ classified spare parts inventories using the Neutrosophic Fuzzy EDAS method to achieve high inventory management efficiency in the aviation industry, where information is often inconsistent and uncertain. Farizal et al.^47^ proposed a novel methodology that blends a modified multi-criteria classification method and a semi-Delphi approach. Khan et al.^48^ proposed a variant of the VIKOR multi criteria decision making approach that incorporates fuzzy set theory to enhance classification flexibility and improve decision making effectiveness. Ayalew et al.^49^ proposed a comprehensive approach that integrates always better control and Vital Essential Desirable (VED) analysis with multi criteria analysis to optimize pharmaceutical inventory control.

In the literature, the concept of mean demand or demand volume is popular. The detailed criteria used in the literature are presented in Table 1 (the other criteria, such as common usage, critical factor, etc., in some papers are integrated into the final column). However, these classification methods do not consider the criterion of demand stability. This paper proposes using mean demand, demand deviation, and demand range to calculate the weight of the demand criterion when classifying inventory items based on demand stability.

**Table 1:** The detailed criteria adopted in existing literature.

References	Methods	Demand			Unit Price	Lead-time	Others
		Mean demand	Standard deviation	Demand range			
Partovi and Anandarajan^29^	AI	√			√	√	√
Kartal et al.^30^	AI	√			√	√	√
Lopez-Soto et al.^31^	AI	√			√	√	√
Cui et al.^32^	AI	√			√	√	√
Qaffas et al.^33^	AI	√				√	√
Yang et al.^13^	AI	√			√	√	√
Ramanathan^7^	DEA-like	√			√	√	
Ng^8^	DEA-like	√			√	√	
Zhou and Fan^14^	DEA-like	√			√	√	
Hadi-Vencheh^15^	DEA-like	√			√	√	
Torabi et al.^25^	DEA-like	√			√	√	√
Hatefi et al.^26^	DEA-like	√			√	√	√
Park et al.^27^	DEA-like	√			√	√	
Cakir and Canbolat^10^	Fuzzy, AHP	√			√	√	√
Chu et al.^16^	ABC, Fuzzy	√			√	√	√
Hadi-Vencheh and Mohamadghasemi^17^	Fuzzy, AHP, DEA	√			√	√	√
Lolli et al.^18^	AHP, K-Veto	√				√	√
Ishizaka et al.^19^	AHP, DEA	√			√		√
Nariswari et al.^20^	AHP	√				√	
Yigit and Esnaf^21^	AHP, FCM-Rveto	√			√	√	√
Rodríguez et al.^22^	Fuzzy, AHP, TOPSIS	√			√		√
Torre et al.^23^	AHP				√	√	
Keshavarz Ghorabaee et al.^41^	EDAS	√			√	√	
Simsek et al.^42^	EDAS	√			√		√
Wu^43^	PIMAR						√
Xu et al.^44^	Group Decision	√			√	√	
Hadi-Vencheh et al.^45^	TOPSIS-AFEA	√			√	√	
Cakmak and Guney^46^	Fuzzy EDAS	√			√		√
Farizal et al.^47^	AHP, Semi-Delphi	√				√	√
Khan et al.^48^	VIKOR, Fuzzy	√			√	√	√
Ayalew et al.^49^	VED	√			√	√	√
The proposed method	DEA-like	√	√	√	√	√	

## Methodology

The method adopted in this paper is based on the Ng-model. To describe the proposed model, the symbols are presented in Table 2.

**Table 2:** Symbols adopted in this paper.

Symbols	Descriptions
*i*	The *i*th item to be evaluated
*I*	Number of items
$j$	The $j$th criterion
$J$	Number of criteria
${y}_{ij}$	The measurement of the *i*th item under the $j$th criterion
${t}_{ij}$	The 0–1 scale score of the *i*th item under the $j$th criterion
${w}_{ij}$	The weight of the *i*th item under the $j$th criterion
${S}_{i}^{\left(1\right)}$	The obtained score of the demand stability of item *i*
${S}_{i}^{\left(2\right)}$	The obtained final score of item *i* considering all criteria
$n$	The $n$th cycle of the historical demand data
$N$	Number of cycles of the historical demand data
${d}_{in}$	The demand size of the *i*th item at cycle $n$
${D}_{i}$	The mean demand of the *i*th item
${\sigma }_{i}$	The standard deviation of the *i*th item demand
${r}_{i}$	The range of the demand of item *i*
${L}_{i}$	The lead-time of item *i*
${P}_{i}$	The unit price of item *i*
${s}_{i}$	The safety stock of item *i*
${Q}_{i}$	Order quantity of item *i*
${FR}_{i}$	Fill rate of item *i*
${FR}_{T}$	The overall fill rate of the inventory system
${k}_{i}$	Safety factor of item *i*
${h}_{i}$	Unit inventory holding cost of item *i*
$\Phi \left(\cdot \right)$	Standard normal probability distribution function
$\mathrm{G}\left(\mathrm{x}\right)$	Loss function of the standard normal distribution
${C}_{T}$	Overall safety stock cost of the inventory system
${CSL}_{i}$	Cycle service level of item *i*

For clarity, the assumptions of the proposed model studied in this paper are listed as follows:

1. The demand of items obeys normal distribution;
2. The continuous review and (s, Q) inventory strategy is utilized.

The Ng-model is a DEA-like model that is efficiently utilized by inventory managers. The model is given by the following linear programming for item *i*:

1 $$\text{Max }{S}_{i}=\sum_{j=1}^{J}{w}_{ij}{y}_{ij}$$

s.t.

2 $$\sum_{j=1}^{J}{w}_{ij}=1$$

3 $${w}_{ij}-{w}_{i\left(j+1\right)}\ge 0,\quad j=1,2, \ldots , \quad \left(J-1\right)$$

4 $${w}_{ij}\ge 0,\quad j= 1,2,\ldots ,J$$

Equation (1) is the objective function that maximizes the weighted sum score of item *i*. Equations (2) and (3) are the constraints that ensure the weights are normalized and rank sequentially. Equation (4) guarantees that the weights are non-negative.

After a transformation, the model is not necessarily solved by a linear optimizer. The value ${max}_{j=\mathrm{1,2},\dots ,J}\left(\frac{1}{j}\sum_{k=1}^{j}{y}_{ik}\right)$ is the final score of item *i*. Note that, considering the constraint (3), inventory managers should rank the criteria in descending order in advance.

The Ng-model first transforms ${y}_{ij}$ to ${t}_{ij}$ by the transformation Eq. (5) proposed by Partovi and Hopton^50^.

5 $${t}_{ij}=\frac{{y}_{ij}-{min}_{i=\mathrm{1,2},\dots ,I}\left\{{y}_{ij}\right\}}{{max}_{i=\mathrm{1,2},\dots ,I}\left\{{y}_{ij}\right\}-{min}_{i=\mathrm{1,2},\dots ,I}\left\{{y}_{ij}\right\}}$$

As to Ng-model, the items are classified by the following process:

- Step 1. Calculate the value of $\frac{1}{j}\sum_{k=1}^{j}{t}_{ik}$ for all items.
- Step 2. The maximum among $\frac{1}{j}\sum_{k=1}^{j}{t}_{ik}$ is the final score of item *i*.
- Step 3. Rank the items by the final score in descending order.
- Step 4. Classify the items by the principle of the ABC classification method.

The Ng-model can be easily implemented in practice. For classifying items considering the demand stability in this paper, the demand criticality scores ${S}_{i}^{\left(1\right)}$ and the final criticality scores ${S}_{i}^{\left(2\right)}$ are calculated successively. The proposed method is called the D-Ng-model, as the two phases of the method are analyzed using the Ng-model.

The D-Ng-model uses three variables, the mean ${D}_{i}$, standard deviation ${\sigma }_{i}$, and range ${r}_{i}$ of demand to describe demand stability. The mean demand presents the overall level of demand, a common variable in articles that consider the demand criterion for MCIC research. The demand standard deviation describes the degree of demand dispersion. Note that, the demand range is another variable that cannot be ignored. Regarding items with similar mean demand and standard deviation, inventory managers should pay closer attention to those with a wider range. It gives rise to the risks of demand forecasting and inventory control. Furthermore, the three variables used to calculate demand stability criticality are all positively related to the importance levels of items, as inventory managers should care more about items with larger demand sizes or more irregular demand patterns. And then, the D-Ng-model combines other criteria to calculate the final criticality scores ${S}_{i}^{\left(2\right)}$. For inventory managers, the process of the D-Ng-model is as follows:

Step 1. Select criteria (e.g., demand, unit price and lead-time) and determine the prioritization of the criteria (${w}_{ij}\ge {w}_{i\left(j+1\right)}$).

Step 2. Input the data of selected criteria, especially calculate the mean demand ${D}_{i}$, the demand standard deviation ${\sigma }_{i}$, and the demand range ${r}_{i}$ of item *i* by Eqs. (6–8);

6 $${D}_{i}=\frac{\sum_{n=1}^{N}{d}_{in}}{N},1\le i\le I$$

7 $${\sigma }_{i}=\sqrt{\frac{\sum_{n=1}^{N}{\left({D}_{i}-{d}_{in}\right)}^{2}}{N-1}}, 1\le i\le I$$

8 $${r}_{i}=\mathrm{max}\left({d}_{in}\right)-\mathrm{min}\left({d}_{in}\right), 1\le i\le I$$

Step 3. Transform ${D}_{i}$, ${\sigma }_{i}$, ${r}_{i}$ and the measurements ${y}_{ij}$ of other criteria to ${t}_{ij}$ (0–1 scale) by Eq. (5);

Step 4. Take ${D}_{i}$, ${\sigma }_{i}$, ${r}_{i}$ as a group of sub-criteria to calculate ${S}_{i}^{\left(1\right)}$ (The criticality scores of the demand stable criterion) by step 2 in the process of Ng-model, i.e., ${S}_{i}^{\left(1\right)}=\mathrm{max}( \frac{1}{j}\sum_{k=1}^{j}{t}_{ik}), j=1,\dots J$.

Step 5. Take the demand stable criterion, unit price and lead-time as a new and final group of criteria to calculate ${S}_{i}^{\left(2\right)}$ (The obtained final score of item *i*) by the same formula in Step 4.

Step 6. Rank the items by the final score ${S}_{i}^{\left(2\right)}$ in descending order and classify the items by ABC principle.

Step 7. Conduct the classification results and make appropriate inventory strategies for each class.

## Results and discussion

### Dataset description

To provide proof of the proposed method, we conduct an empirical investigation. The MCIC case study considering demand stability is from a manufacturer in China (China Railway Engineering Equipment Group Corporation). The company fabricates tunnel boring machines and offers after-sales service. The sales products are across China. The company maintains and repairs the sold machines and must reserve spare parts for repairing failed products. The SKUs are numerous due to product complexity and multiple product versions. The warehouses of spare parts are distributed to offer efficient after-sales service. The supply of spare parts is essential for under-warranty service, which can increase customer satisfaction. In addition, the spare parts can be recognized as commodities for out-of-warranty service, which can be a source of economic benefit for the company.

The sales volume of tunnel boring machines cannot be accurately forecast due to national policies and competitive factors. In addition, the company is not well acquainted with the operating times and working conditions of the sold-out tunnel boring machines in the country. Furthermore, the causes of tunnel boring machine failures are complex, and maintenance policies may vary. According to some maintenance strategies, failed components should be replaced with spare parts, but they can also be repaired using other strategies. These factors make spare parts demand unstable and unpredictable. Adequate spare parts inventory is necessary to ensure customer satisfaction.

It is troublesome for spare parts inventory managers in the company to efficiently manage the diverse spare parts whose demand is unstable. The current inventory strategies are mostly experiential. The inventory managers pay close attention to high-value or high-demand spare parts. Nevertheless, it is common for some spare parts to be out of stock, while others are overstocked or even obsolete.

To assist inventory managers in efficiently managing spare parts inventories and to conduct empirical analysis of MCIC considering demand stability, a dataset of 52 items is presented as a case study. The MCIC results for the 52 items are generated by the D-Ng-model and Ng-model. The dataset presents monthly demand data from January to December 2022. The historical demand, unit price, and lead-time for the 52 items are shown in Table 3. The units of criteria are listed in Table 4.

**Table 3:** The measurements of demand, unit price, and lead-time of items.

Item no	JAN	FEB	MAR	APR	MAY	JUN	JUL	AUG	SEP	OCT	NOV	DEC	Lead-time	Price (￥)
1	422	453	530	64	92	349	64	64	453	74	247	142	1	150.80
2	733	523	349	631	347	460	213	555	345	99	54	235	1	484.69
3	546	332	95	84	212	355	327	240	702	235	302	124	3	635.88
4	329	463	322	204	320	764	546	781	463	649	641	439	2	216.05
5	355	743	632	281	740	214	733	462	453	214	220	425	2	117.94
6	235	463	674	653	234	463	755	735	235	545	235	751	1	142.38
7	343	547	392	784	566	574	321	450	579	433	135	75	3	429.08
8	452	322	542	523	454	289	742	450	346	469	754	349	3	387.85
9	289	754	568	435	566	671	506	76	457	75	424	196	2	489.64
10	325	346	645	456	453	564	765	854	561	340	650	201	2	216.05
11	782	453	1093	740	544	654	198	554	550	753	532	601	5	715.21
12	833	1034	1093	340	461	520	436	1093	601	654	769	872	3	194.44
13	893	671	902	340	801	393	610	874	924	845	750	530	3	216.05
14	98	99	103	343	104	130	357	305	302	913	382	578	4	265.22
15	878	265	701	346	601	435	260	361	203	342	165	750	2	198.98
16	345	853	302	345	655	832	509	641	235	765	409	450	4	905.24
17	734	445	700	435	502	435	830	324	563	722	359	461	2	150.74
18	433	659	450	453	594	239	457	120	282	230	351	112	4	413.52
19	873	923	834	903	340	777	348	660	310	392	993	210	2	272.52
20	873	1009	239	301	739	789	560	805	874	880	490	378	3	168.75
21	326	632	840	298	450	140	125	169	630	690	460	246	4	439.90
22	873	235	982	745	938	732	745	634	856	644	450	598	1	105.19
23	237	872	630	572	733	310	834	889	388	529	230	1081	3	413.52
24	459	889	347	783	934	774	821	934	850	620	661	927	2	298.90
25	930	994	340	813	342	877	950	642	590	758	672	539	2	122.04
26	775	647	456	885	647	450	572	582	590	450	420	594	4	124.61
27	194	103	192	99	124	193	100	230	284	40	255	390	3	529.07
28	775	647	456	885	647	450	572	582	590	450	420	594	5	684.33
29	883	934	949	845	773	610	788	821	924	1004	699	239	3	413.50
30	395	596	303	490	140	309	310	435	320	560	235	459	4	453.37
31	340	750	1200	405	349	294	692	693	654	520	791	620	4	121.99
32	450	602	569	799	404	388	569	459	503	596	452	369	4	191.06
33	102	349	699	934	685	592	568	794	595	270	456	992	2	169.99
34	390	238	499	450	291	349	692	109	542	873	294	569	2	113.61
35	249	598	983	451	390	998	392	562	204	640	420	884	5	528.08
36	948	510	670	922	620	710	570	994	300	502	549	1059	4	331.31
37	745	945	745	453	281	840	414	651	845	340	703	392	3	1045.70
38	543	502	103	482	593	203	1049	922	592	759	328	780	4	292.43
39	194	114	300	103	683	310	193	68	239	384	688	200	5	564.45
40	439	938	993	1095	884	783	600	283	692	601	593	596	4	303.11
41	390	488	821	424	862	469	963	194	833	923	691	590	3	767.61
42	433	1052	699	617	880	723	841	434	868	762	850	583	4	305.84
43	290	739	195	832	591	983	887	613	459	880	723	624	4	561.20
44	124	620	723	922	459	194	582	189	953	381	712	478	5	757.28
45	382	965	986	922	903	348	746	549	512	743	433	104	2	291.13
46	193	993	204	205	383	389	104	822	388	491	583	592	3	284.99
47	764	820	459	195	593	692	592	951	358	932	359	104	2	320.99
48	192	239	831	299	292	349	92	91	349	200	450	74	4	352.52
49	352	795	903	952	983	508	591	544	820	463	433	103	4	343.77
50	204	533	924	103	359	500	200	177	452	495	920	742	4	380.26
51	293	102	883	812	402	357	992	452	712	492	149	592	4	668.04
52	102	993	340	249	631	593	640	219	732	525	815	752	3	386.77

**Table 4:** The units of criteria.

Criteria	Units
Demands	Month
Lead time	Month
Price	CNY

### Classification results

By step 1 and step 2 in the D-Ng-model, ${\mathrm{D}}_{\mathrm{i}}$, ${\upsigma }_{\mathrm{i}}$, ${\mathrm{r}}_{\mathrm{i}}$, and ${\mathrm{t}}_{\mathrm{ij}}$ are calculated and shown in Table 5. By step 3, the sequence of criterion weights in descending order is the mean, standard deviation and range of demand. Similarly, we make a descending sequence, i.e., the demand criterion, the unit price, and the lead-time in step 4. After calculating the final scores ${\mathrm{S}}_{\mathrm{i}}^{\left(2\right)}$, the items are ranked in descending order in step 5. In the final step, the same distribution of class A, B, and C items by Ng^8^ is adopted, i.e., 10 items in class A, 16 items in class B, and 26 items in class C to make a comparison. The calculated results from step 3 to step 6 are shown in Table 6. The items sequence in Table 6 has been rearranged according to the classification results by D-Ng-model.

**Table 5:** The results of calculating ${D}_{i}$, ${\sigma }_{i}$, ${r}_{i}$ and ${t}_{ij}$.

Item no	${D}_{i}$	${\sigma }_{i}$	${r}_{i}$	${D}_{i}$(0–1 scale)	${\sigma }_{i}$(0–1 scale)	${r}_{i}$(0–1 scale)	Price (0–1 scale)	Lead Time (0–1 scale)
1	246.17	183.76	466	0.10	0.45	0.19	0.05	0.00
2	378.67	209.43	679	0.32	0.59	0.55	0.40	0.00
3	296.17	181.40	618	0.19	0.44	0.45	0.56	0.50
4	493.42	185.80	577	0.51	0.46	0.38	0.12	0.25
5	456.00	210.06	529	0.45	0.59	0.30	0.01	0.25
6	498.17	218.07	521	0.52	0.63	0.29	0.04	0.00
7	433.25	198.78	709	0.41	0.53	0.60	0.34	0.50
8	474.33	150.17	465	0.48	0.28	0.19	0.30	0.50
9	418.08	219.80	679	0.39	0.64	0.55	0.41	0.25
10	513.33	194.99	653	0.54	0.51	0.51	0.12	0.25
11	621.17	215.81	895	0.72	0.62	0.91	0.65	1.00
12	725.50	263.79	753	0.89	0.87	0.68	0.09	0.50
13	711.08	202.81	584	0.87	0.55	0.39	0.12	0.50
14	309.50	243.30	815	0.21	0.76	0.78	0.17	0.75
15	442.25	233.83	713	0.43	0.71	0.61	0.10	0.25
16	528.42	214.82	618	0.57	0.61	0.45	0.85	0.75
17	542.50	164.89	506	0.59	0.35	0.26	0.05	0.25
18	365.00	173.77	547	0.30	0.40	0.33	0.33	0.75
19	630.25	288.32	783	0.74	1.00	0.73	0.18	0.25
20	661.42	257.58	770	0.79	0.84	0.70	0.07	0.50
21	417.17	238.02	715	0.39	0.74	0.61	0.36	0.75
22	702.67	211.50	747	0.86	0.60	0.67	0.00	0.00
23	608.75	280.39	851	0.70	0.96	0.84	0.33	0.50
24	749.92	192.35	587	0.94	0.50	0.40	0.21	0.25
25	703.92	223.41	654	0.86	0.66	0.51	0.02	0.25
26	589.00	139.54	465	0.67	0.22	0.19	0.02	0.75
27	183.67	97.71	350	0.00	0.00	0.00	0.45	0.50
28	589.00	139.54	465	0.67	0.22	0.19	0.62	1.00
29	789.08	206.04	765	1.00	0.57	0.70	0.33	0.50
30	379.33	134.41	456	0.32	0.19	0.18	0.37	0.75
31	609.00	253.54	906	0.70	0.82	0.93	0.02	0.75
32	513.33	121.29	430	0.54	0.12	0.13	0.09	0.75
33	586.33	262.75	890	0.67	0.87	0.91	0.07	0.25
34	441.33	210.25	764	0.43	0.59	0.69	0.01	0.25
35	564.25	268.91	794	0.63	0.90	0.74	0.45	1.00
36	696.17	235.17	759	0.85	0.72	0.69	0.24	0.75
37	612.83	225.38	664	0.71	0.67	0.53	1.00	0.50
38	571.33	279.38	946	0.64	0.95	1.00	0.20	0.75
39	289.67	205.99	620	0.18	0.57	0.45	0.49	1.00
40	708.08	237.91	812	0.87	0.74	0.78	0.21	0.75
41	637.33	246.64	769	0.75	0.78	0.70	0.70	0.50
42	728.50	186.88	619	0.90	0.47	0.45	0.21	0.75
43	651.33	242.14	788	0.77	0.76	0.73	0.48	0.75
44	528.08	275.96	829	0.57	0.94	0.80	0.69	1.00
45	632.75	286.34	882	0.74	0.99	0.89	0.20	0.25
46	445.58	268.14	889	0.43	0.89	0.90	0.19	0.50
47	568.25	278.27	847	0.64	0.95	0.83	0.23	0.25
48	288.17	206.94	757	0.17	0.57	0.68	0.26	0.75
49	620.58	271.46	880	0.72	0.91	0.89	0.25	0.75
50	467.42	280.40	821	0.47	0.96	0.79	0.29	0.75
51	519.83	284.71	890	0.56	0.98	0.91	0.60	0.75
52	549.25	270.10	891	0.60	0.90	0.91	0.30	0.50
Max	789.08	288.32	946					
Min	183.67	97.71	350

**Table 6:** The final classification results of the D-Ng-model and Ng-model.

Item no	${S}_{i}^{\left(1\right)}$ of D-Ng-model	${S}_{i}^{\left(2\right)}$ of D-Ng-model	The scores of Ng-model	ABC classification	
				D-Ng-model	Ng-model
12	0.89	0.89	0.89	A	A
13	0.87	0.87	0.87	A	A
19	0.87	0.87	0.74	A	B
24	0.94	0.94	0.94	A	A
25	0.86	0.86	0.86	A	A
29	1.00	1.00	1.00	A	A
38	0.86	0.86	0.64	A	B
40	0.87	0.87	0.87	A	A
42	0.90	0.90	0.90	A	A
45	0.87	0.87	0.74	A	B
11	0.75	0.80	0.79	B	B
20	0.81	0.81	0.79	B	B
22	0.86	0.86	0.86	B	A
23	0.83	0.83	0.70	B	B
31	0.82	0.82	0.70	B	B
33	0.81	0.81	0.67	B	B
35	0.76	0.76	0.69	B	B
36	0.85	0.85	0.85	B	A
37	0.71	0.85	0.85	B	A
41	0.77	0.77	0.75	B	B
43	0.77	0.77	0.77	B	B
44	0.77	0.82	0.75	B	B
47	0.81	0.81	0.64	B	C
49	0.84	0.84	0.72	B	B
51	0.81	0.81	0.63	B	C
52	0.81	0.81	0.60	B	C
1	0.28	0.28	0.10	C	C
2	0.49	0.49	0.36	C	C
3	0.36	0.47	0.42	C	C
4	0.51	0.51	0.51	C	C
5	0.52	0.52	0.45	C	C
6	0.58	0.58	0.52	C	C
7	0.51	0.51	0.42	C	C
8	0.48	0.48	0.48	C	C
9	0.53	0.53	0.40	C	C
10	0.54	0.54	0.54	C	C
14	0.58	0.58	0.38	C	C
15	0.58	0.58	0.43	C	C
16	0.59	0.73	0.72	C	B
17	0.59	0.59	0.59	C	C
18	0.35	0.48	0.46	C	C
21	0.58	0.58	0.50	C	C
26	0.67	0.67	0.67	C	B
27	0.00	0.32	0.32	C	C
28	0.67	0.76	0.76	C	B
30	0.32	0.48	0.48	C	C
32	0.54	0.54	0.54	C	C
34	0.57	0.57	0.43	C	C
39	0.40	0.63	0.55	C	C
46	0.74	0.74	0.43	C	C
48	0.48	0.50	0.40	C	C
50	0.74	0.74	0.50	C	C

The classification results in Table 6 show the changes resulting from considering demand stability. The D-Ng-model demotes some items in class A to class B while some items in class B are promoted to class A, etc. For example, although the mean demand of items 19, 38, and 45 is lower, the measurements of the demand standard deviation are higher, so the items are classified into class A by the D-Ng-model, but into class B by the Ng-model. Although the final scores for items 22, 36, and 37 are identical across the two models, the three items are demoted to class B by the D-Ng-model due to the rearrangement.

### The inventory performance of classification results

This paper adopts the same inventory performance evaluation method and inventory systems proposed by Babai et al.^24^ to compare inventory performance under the D-Ng-model with that under the Ng-model.

The ${C}_{T}$ can be calculated by Eq. (9), which references Babai et al.^24^.

9 $${C}_{T}=\sum_{i=1}^{I}{h}_{i}{k}_{i}{\sigma }_{i}\sqrt{{L}_{i}}$$

where the safety factor ${k}_{i}$ of each item *i* is given by:

10 $${k}_{i}={\Phi }^{-1}\left({CSL}_{i}\right)$$

The ${FR}_{T}$ is the weighted average calculated by Eq. (11).

11 $${FR}_{T}=\frac{\sum_{i=1}^{I}{FR}_{i}{D}_{i}}{\sum_{i=1}^{I}{D}_{i}}$$

where the fill rate of each item *i* is given by:

12 $${FR}_{i}=1-\frac{{\sigma }_{i}\sqrt{{L}_{i}}}{{Q}_{i}}G({k}_{i})$$

And

13 $$G\left({k}_{i}\right)=\frac{1}{\sqrt{2\pi }}{e}^{-\frac{{k}_{i}^{2}}{2}}-{k}_{i}[1-\Phi \left({k}_{i}\right)]$$

**Fig. 1:**
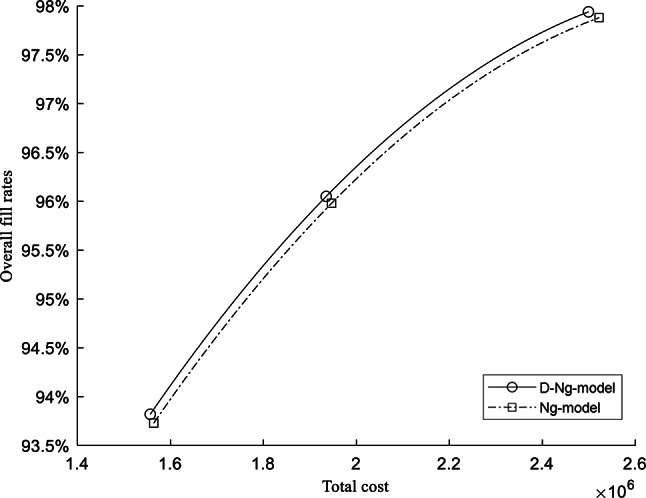
The total cost and overall fill rates by D-Ng-model and Ng-model.

Assume that ${h}_{i}$ is to be 20% of ${P}_{i}$. The dataset has been verified that the demand corresponds to normal distribution. As the manufacturer purchases spare parts monthly and the ordering cost is not readily available, we assume that this is the average monthly demand. The detailed calculated results for the inventory performance are presented in Table 7 and Fig. 1, with CSLs of 99%, 95%, and 90% for classes A, B, and C, respectively. The total safety stock inventory cost for the D-Ng-model and Ng-model is 2,499,346.83 ¥ and 2,522,108.07 ¥, respectively. Correspondingly, the overall fill rates are 97.94% and 97.88%, respectively. The difference is 22,761.24 ¥ decrease in total cost and 0.06% increase in overall fill rate.

**Table 7:** The detailed results of inventory performance when CSLs are (99%, 95%, 90%).

Item no	D-Ng-model				Ng-model			
	Class	${CSL}_{i}$	${FR}_{i}$	Item cost	Class	${CSL}_{i}$	${FR}_{i}$	Item Cost
1	C	0.90	96.47%	7102.45	C	0.90	96.47%	7102.45
2	C	0.90	97.38%	26,018.21	C	0.90	97.38%	26,018.21
3	C	0.90	94.98%	51,207.18	C	0.90	94.98%	51,207.18
4	C	0.90	97.48%	14,550.55	C	0.90	97.48%	14,550.55
5	C	0.90	96.92%	8980.27	C	0.90	96.92%	8980.27
6	C	0.90	97.93%	7958.05	C	0.90	97.93%	7958.05
7	C	0.90	96.24%	37,865.67	C	0.90	96.24%	37,865.67
8	C	0.90	97.40%	25,857.22	C	0.90	97.40%	25,857.22
9	C	0.90	96.48%	39,011.04	C	0.90	96.48%	39,011.04
10	C	0.90	97.46%	15,270.39	C	0.90	97.46%	15,270.39
11	B	0.95	98.38%	113,539.41	B	0.95	98.38%	113,539.41
12	A	0.99	99.79%	41,333.69	A	0.99	99.79%	41,333.69
13	A	0.99	99.83%	35,310.76	A	0.99	99.83%	35,310.76
14	C	0.90	92.56%	33,078.58	C	0.90	92.56%	33,078.58
15	C	0.90	96.46%	16,865.30	C	0.90	96.46%	16,865.30
16	C	0.90	96.15%	99,685.20	B	0.95	98.30%	127,944.57
17	C	0.90	97.96%	9009.80	C	0.90	97.96%	9009.80
18	C	0.90	95.49%	36,835.45	C	0.90	95.49%	36,835.45
19	A	0.99	99.78%	51,699.58	B	0.95	98.65%	36,554.40
20	B	0.95	98.59%	24,766.56	B	0.95	98.59%	24,766.56
21	C	0.90	94.60%	53,673.12	C	0.90	94.60%	53,673.12
22	B	0.95	99.37%	7318.75	A	0.99	99.90%	10,351.04
23	B	0.95	98.33%	66,066.84	B	0.95	98.33%	66,066.84
24	A	0.99	99.88%	37,829.90	A	0.99	99.88%	37,829.90
25	A	0.99	99.85%	17,940.32	A	0.99	99.85%	17,940.32
26	C	0.90	97.76%	8913.71	B	0.95	99.01%	11,440.62
27	C	0.90	95.64%	22,949.25	C	0.90	95.64%	22,949.25
28	C	0.90	97.49%	54,730.07	B	0.95	98.89%	70,245.29
29	A	0.99	99.85%	68,657.58	A	0.99	99.85%	68,657.58
30	C	0.90	96.64%	31,238.60	C	0.90	96.64%	31,238.60
31	B	0.95	98.26%	20,349.59	B	0.95	98.26%	20,349.59
32	C	0.90	97.76%	11,879.33	C	0.90	97.76%	11,879.33
33	B	0.95	98.68%	20,779.40	B	0.95	98.68%	20,779.40
34	C	0.90	96.81%	8658.36	C	0.90	96.81%	8658.36
35	B	0.95	97.77%	104,459.99	B	0.95	97.77%	104,459.99
36	B	0.95	98.59%	51,262.10	A	0.99	99.77%	72,500.97
37	B	0.95	98.67%	134,289.71	A	0.99	99.78%	189,928.50
38	A	0.99	99.67%	76,024.72	B	0.95	97.96%	53,753.59
39	C	0.90	92.47%	66,637.54	C	0.90	92.47%	66,637.54
40	A	0.99	99.77%	67,104.21	A	0.99	99.77%	67,104.21
41	B	0.95	98.60%	107,875.34	B	0.95	98.60%	107,875.34
42	A	0.99	99.83%	53,184.87	A	0.99	99.83%	53,184.87
43	B	0.95	98.45%	89,406.51	B	0.95	98.45%	89,406.51
44	B	0.95	97.56%	153,724.39	B	0.95	97.56%	153,724.39
45	A	0.99	99.78%	54,851.21	B	0.95	98.66%	38,782.77
46	C	0.90	95.07%	33,924.92	C	0.90	95.07%	33,924.92
47	B	0.95	98.55%	41,555.47	C	0.90	96.72%	32,377.03
48	C	0.90	93.20%	37,395.44	C	0.90	93.20%	37,395.44
49	B	0.95	98.17%	61,398.93	B	0.95	98.17%	61,398.93
50	C	0.90	94.32%	54,657.72	C	0.90	94.32%	54,657.72
51	B	0.95	97.71%	125,139.08	C	0.90	94.81%	97,499.36
52	B	0.95	98.22%	59,524.51	C	0.90	95.97%	46,377.21
			97.94%	2,499,346.83			97.88%	2,522,108.07

The results of inventory performance, including the CSLs of (95%, 90%, 85%) and (90%, 85%, 80%), are shown in Table 8. The D-Ng-model still shows better inventory performance. The differences of ${FR}_{T}$ and ${C}_{T}$ in Table 5 is the subtraction from the calculated results of D-Ng-model to the ones of Ng-model. The results indicate that the difference in overall fill rate is small. This is because the fill rates of different CSLs are already high. Nevertheless, the cost decrease with CSLs is visible.

**Table 8:** The results of inventory performance under three groups of CSLs combinations.

CSLs (A, B, C)	D-Ng-model		Ng-model		Diff	
	${FR}_{T}$	${C}_{T}$(¥)	${FR}_{T}$	${C}_{T}$(¥)	${FR}_{T}$	${C}_{T}$(¥)
(99%, 95%, 90%)	97.94%	2,499,346.83	97.88%	2,522,108.07	0.06%	− 22,761.24
(95%, 90%, 85%)	96.05%	1,935,087.68	95.98%	1,946,702.81	0.07%	− 11,615.13
(90%, 85%, 80%)	93.82%	1,556,595.22	93.73%	1,564,135.06	0.07%	− 7539.84

According to the results, in general, the D-Ng-model performs better across different CSLs. This analysis indicates that the company can achieve better inventory performance at a lower cost by prioritizing demand stability.

### The sensitivity analysis of ${{{Q}}}_{{{i}}}$

It is common that the parameters ${\sigma }_{i}$, ${L}_{i}$ and ${k}_{i}$ remain constant over a certain period of time. For inventory managers, they are also not straightforward to be manipulated or regulated. Compared with ${\sigma }_{i}$, ${L}_{i}$ and ${k}_{i}$, the order quantity ${Q}_{i}$ is regularly modified to align with diverse inventory strategies in practice. For further analysis and proposing rational inventory strategies for inventory managers, we have adjusted the rate of ${Q}_{i}$ to monthly demand average from 0.2 to 10.1 under three different CSLs combinations. Note that the step is 0.1 when the rate is 0.2–1.1, while the step is 1 when the rate is 1.1–10.1. The reason of step set is that the change of ${FR}_{T}$ is very small when the ${Q}_{i}$ is high-level. Thus, the change can be explicitly observed by larger step. It is apparent that the inventory holding cost become higher with the decreasing of ${Q}_{i}$. Thus, the ${FR}_{T}$ is mainly discussed in this analysis.

**Fig. 2:**
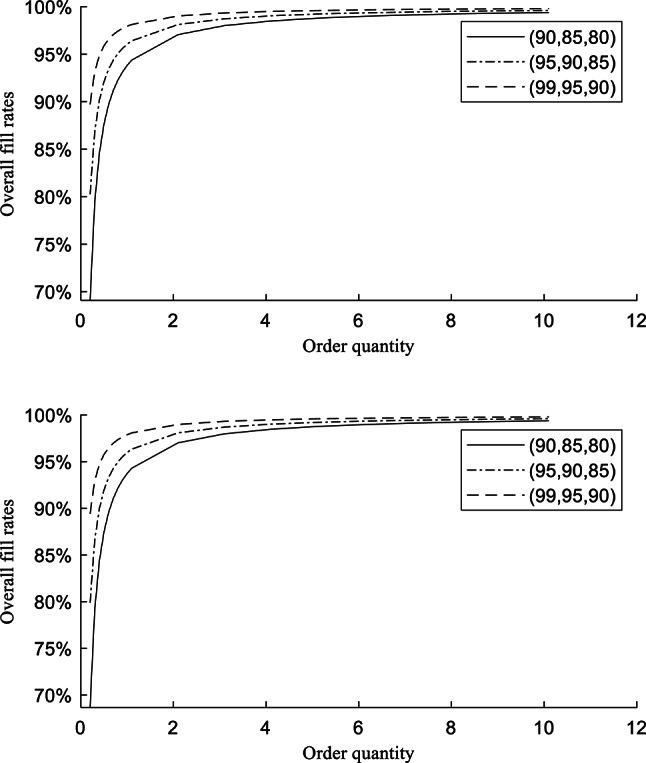
The overall fill rates with order quantity by D-Ng-model and Ng-model.

**Fig. 3:**
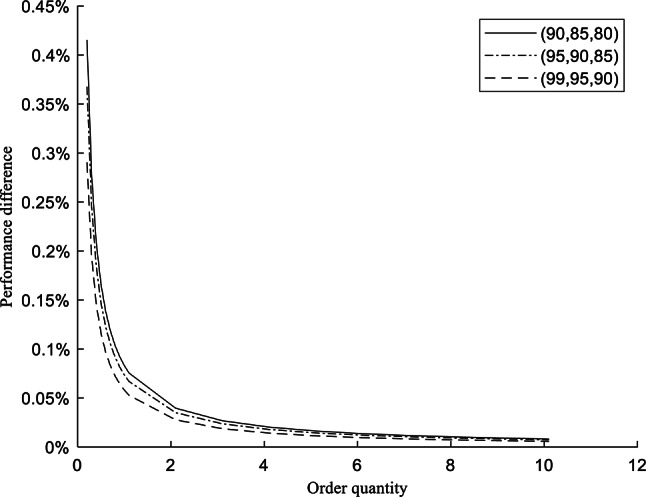
The performance differences with order quantity between models.

The calculated results of ${FR}_{T}$ by D-Ng-model and Ng-model under (99%, 95%, 90%), (95%, 90%, 85%), and (90%, 85%, 80%) CSLs are respectively presented in Table 9 and Fig. 2. For explicitly observing the changes between these results, the differences are calculated and presented in Table 10 and Fig. 3. The differences between models with the same rate of ${Q}_{i}$ and CSLs are the subtraction from the calculated results of D-Ng-model to the ones of Ng-model, which is same in Table 8. The differences between adjacent rates of ${Q}_{i}$ with the same CSLs and by the same model are the subtraction from the calculated results of higher rates to the ones of lesser rates.

**Table 9:** The results of ${FR}_{T}$ by adjusting ${Q}_{i}$.

Rate of ${Q}_{i}$	(90%, 85%, 80%)		(95%, 90%, 85%)		(99%, 95%, 90%)	
	${FR}_{T}$(D-Ng-Model)	${FR}_{T}$(Ng-model)	${FR}_{T}$(D-Ng-Model)	${FR}_{T}$(Ng-model)	${FR}_{T}$(D-Ng-Model)	${FR}_{T}$(Ng-model)
0.2	69.0893%	68.6740%	80.2604%	79.8928%	89.7152%	89.4247%
0.3	79.3929%	79.1160%	86.8403%	86.5952%	93.1434%	92.9498%
0.4	84.5447%	84.3370%	90.1302%	89.9464%	94.8576%	94.7124%
0.5	87.6357%	87.4696%	92.1042%	91.9571%	95.8861%	95.7699%
0.6	89.6964%	89.5580%	93.4201%	93.2976%	96.5717%	96.4749%
0.7	91.1684%	91.0497%	94.3601%	94.2551%	97.0615%	96.9785%
0.8	92.2723%	92.1685%	95.0651%	94.9732%	97.4288%	97.3562%
0.9	93.1310%	93.0387%	95.6134%	95.5317%	97.7145%	97.6499%
1.0	93.8179%	93.7348%	96.0521%	95.9786%	97.9430%	97.8849%
1.1	94.3799%	94.3044%	96.4110%	96.3441%	98.1300%	98.0772%
2.1	97.0561%	97.0166%	98.1200%	98.0850%	99.0205%	98.9928%
3.1	98.0058%	97.9790%	98.7265%	98.7028%	99.3365%	99.3177%
4.1	98.4922%	98.4719%	99.0371%	99.0192%	99.4983%	99.4841%
5.1	98.7878%	98.7715%	99.2259%	99.2115%	99.5967%	99.5853%
6.1	98.9865%	98.9729%	99.3528%	99.3407%	99.6628%	99.6533%
7.1	99.1293%	99.1176%	99.4440%	99.4336%	99.7103%	99.7021%
8.1	99.2368%	99.2265%	99.5126%	99.5035%	99.7461%	99.7389%
9.1	99.3206%	99.3115%	99.5662%	99.5581%	99.7740%	99.7676%
10.1	99.3879%	99.3797%	99.6091%	99.6018%	99.7963%	99.7906%

**Table 10:** The differences between models and adjacent rates of ${Q}_{i}$.

Rate of ${Q}_{i}$	(90%, 85%, 80%)			(95%, 90%, 85%)			(99%, 95%, 90%)		
	${FR}_{T}$ diff. between models	${FR}_{T}$ diff. with adjacent ${Q}_{i}$		${FR}_{T}$ diff. between models	${FR}_{T}$ diff. with adjacent ${Q}_{i}$		${FR}_{T} d$ iff. between models	${FR}_{T}$ diff. with adjacent ${Q}_{i}$	
		D-Ng-model	Ng-model		D-Ng-model	Ng-model		D-Ng-model	Ng-model
0.2	0.4154%	–	–	0.3677%	–	–	0.2905%	–	–
0.3	0.2769%	10.3036%	10.4420%	0.2451%	6.5799%	6.7024%	0.1936%	3.4283%	3.5251%
0.4	0.2077%	5.1518%	5.2210%	0.1838%	3.2899%	3.3512%	0.1452%	1.7141%	1.7625%
0.5	0.1661%	3.0911%	3.1326%	0.1471%	1.9740%	2.0107%	0.1162%	1.0285%	1.0575%
0.6	0.1385%	2.0607%	2.0884%	0.1226%	1.3160%	1.3405%	0.0968%	0.6857%	0.7050%
0.7	0.1187%	1.4719%	1.4917%	0.1051%	0.9400%	0.9575%	0.0830%	0.4898%	0.5036%
0.8	0.1038%	1.1040%	1.1188%	0.0919%	0.7050%	0.7181%	0.0726%	0.3673%	0.3777%
0.9	0.0923%	0.8586%	0.8702%	0.0817%	0.5483%	0.5585%	0.0645%	0.2857%	0.2938%
1.0	0.0831%	0.6869%	0.6961%	0.0735%	0.4387%	0.4468%	0.0581%	0.2286%	0.2350%
1.1	0.0755%	0.5620%	0.5696%	0.0669%	0.3589%	0.3656%	0.0528%	0.1870%	0.1923%
2.1	0.0396%	2.6762%	2.7122%	0.0350%	1.7091%	1.7409%	0.0277%	0.8905%	0.9156%
3.1	0.0268%	0.9496%	0.9624%	0.0237%	0.6064%	0.6177%	0.0187%	0.3160%	0.3249%
4.1	0.0203%	0.4864%	0.4929%	0.0179%	0.3106%	0.3164%	0.0142%	0.1618%	0.1664%
5.1	0.0163%	0.2957%	0.2996%	0.0144%	0.1888%	0.1923%	0.0114%	0.0984%	0.1012%
6.1	0.0136%	0.1987%	0.2014%	0.0121%	0.1269%	0.1293%	0.0095%	0.0661%	0.0680%
7.1	0.0117%	0.1427%	0.1447%	0.0104%	0.0912%	0.0929%	0.0082%	0.0475%	0.0488%
8.1	0.0103%	0.1075%	0.1089%	0.0091%	0.0686%	0.0699%	0.0072%	0.0358%	0.0368%
9.1	0.0091%	0.0839%	0.0850%	0.0081%	0.0536%	0.0546%	0.0064%	0.0279%	0.0287%
10.1	0.0082%	0.0673%	0.0682%	0.0073%	0.0430%	0.0438%	0.0058%	0.0224%	0.0230%

Table 9 illustrates that the D-Ng-model still gets better inventory performance than the Ng-model. In Table 10, overall, the differences between models and adjacent rates are the most obvious under the lowest (90%, 85%, 80%) CSLs. The decreasing range of ${FR}_{T}$ gets slowly with the increasing rates of ${Q}_{i}$, and the inventory holding cost will gets higher. This means that the inventory managers should not exceedingly pursue higher fill rates of inventory.

### Managerial implications

The classification results have been significantly changed as inventory managers applied the D-Ng-model. These classification results will assist inventory managers in developing appropriate inventory strategies for SKUs with unstable demand. Since SKUs with unstable demand are more likely to be assigned to higher-importance categories, inventory managers can implement lean inventory management for them. The inventory performance analysis of classification results indicates that fill rates increase while costs decrease.

For the sensitivity analysis of order quantity, the inventory performance results indicate that the D-Ng-model outperforms the Ng-model across various order quantity scenarios. The results also show that increasing the order quantity can raise the fill rate, but the rate of improvement gradually slows. In practice, increasing the order quantity results in higher ordering and holding costs. If SKUs become obsolete, serious inventory obsolescence costs will be incurred. Therefore, inventory managers should not blindly pursue extremely high fill rates.

The proposed model can improve inventory performance. Furthermore, in practical production, enhancing demand forecasting accuracy and shortening spare parts lead-time are of significant importance for boosting inventory performance, especially for SKUs characterized by unstable demand and long lead-time.

## Conclusion

This paper argues that demand stability should be considered when using the demand criterion for MCIC. A case study of a tunnel boring machine manufacturer in China is presented for empirical investigation. The company’s inventory managers face unstable inventory demand. A two-phase method, the D-Ng-model, based on the Ng-model, is proposed for MCIC. Then, a dataset of 52 items in a warehouse is used to verify the D-Ng-model and compare its MCIC results with those of the Ng-model. The results indicate that the D-Ng-model adjusts the criticality sequence of items because of the accessorial demand stability criterion. This adjustment could draw the attention of inventory managers to items with erratic demand characteristics. The calculated results indicate that MCIC results, considering demand stability, yield higher overall fill rates than the Ng-model while incurring lower costs. The case study indicates that MCIC, considering demand stability, could improve inventory performance.

Regarding the limitations of the D-Ng model, the selection and prioritization of criteria are determined subjectively. Theunissen et al.^6^ suggest that AI can be applied to mitigate the risk of subjectivity. To improve the proposed method, the AI method can serve as an alternative for selecting and prioritizing criteria. Furthermore, Song et al.^51^, Wang et al.^52^ and Ducharme et al.^53^ have noted that demand intermittency is a characteristic that undermines forecast accuracy and, consequently, inventory management. This further suggests that demand intermittency could be considered as an additional criterion in future research on multi-criteria inventory criticality for inventory classification.

## Data Availability

The data that support the findings of this study are openly available in Open Science Framework at 10.17605/OSF.IO/Q83D4.

## Abbreviations

MCIC: Multi-criteria inventory classification
SKU: Stock keeping unit
D-Ng-model: Double Ng-model
AHP: Analytic hierarchy process
DEA: Data envelopment analysis
AI: Artificial intelligence
ANN: Artificial neural networks
GA: Genetic algorithms
SVM: Support vector machine
EDAS: Evaluation based on distance from average solution
PIMAR: Parsimoniously interactive multi-attribute rating
TOPSIS-AFEA: TOPSIS-alternative factor extraction approaches
VED: Vital essential desirable
